# 

**Published:** 1889-01

**Authors:** 


THE MEDICAL SOCIETY OF THE STATE OF NEW YORK will hold its next annual meeting in Albany, on Tuesday, Wednesday, and Thursday, February 5, 6, and 7, 1889. All members of county societies in good standing, throughout the State, are invited to attend. Visitors from other States will be cordially welcome. We hoped to publish the preliminary programme in this number, but it appears not to be ready.

THE MEDICAL SOCIETY OF ERIE COUNTY will hold its regular annual meeting on Tuesday, January 8, 1889, in the Y. M. C. A. building, corner West Mohawk and Pearl streets. Election of officers for the ensuing year, and of delegates to the State Medical Society for three years.

THE SOUTHERN SURGICAL AND GYNECOLOGICAL ASSOCIATION held a most successful meeting in Birmingham last month. Dr. Hunter McGuire, of Richmond, Va., was elected President for 1889—an earnest of continued good work.

THE BUFFALO MEDICAL AND SURGICAL ASSOCIATION will hold its regular monthly meetings, 1889, at the Mechanics' Institute, No. 9 Mohawk street.

### BOOKS AND PAMPHLETS RECEIVED.

Lectures on Ectopic Pregnancy and Pelvic Hematocele. By Lawson Tait, F. R. C. S., Edin. and Eng., LL. D., M. D. (Honoris causâ), of the University of New York, Union University of Albany, and the College of Physicians and Surgeons of St. Louis; Professor of Gynecology in Queen's College, Birmingham, etc. Birmingham: The "Journal" Printing Works, New street. 1888.

Leçons de Gynécologie Opératoire. Par Vulliet, Professeur a la Faculté de Medecine de Genève, etc., and Lutaud, Professeur libre de Gynécologie à l'Ecole Pratique, etc. Avec 180 figures intercalés dans le texte. Paris: Librairie J. B. Bailliére et Fils, 19 Rue Hautefeuille. 1889.

The American System of Gynecology. Vol. II. Edited by M. D. Mann, M. D. Pp. xvi., 1180. Four colored plates, 361 wood engravings. Lea Brothers & Co. 1888.

The Efficacy of Coca Erythroxylon. Notes and Comments by Prominent Physicians. Second edition. Mariani, Paris and New York.

The Physician's Leisure Library. Hysteria and Epilepsy. By J. Leonard Corning, M. D., Consultant in Nervous Diseases to St. Francis Hospital, etc., etc. Detroit: George S. Davis. 1888.

Favorite Prescriptions of Distinguished Practitioners, with Notes on Treatment. Compiled from the published writings or unpublished records of Drs. Fordyce Barker, Roberts Bartholow, Samuel D. Gross, A. Jacobi, W. A. Hammond, etc., etc. By B. W. Palmer, A. M., M. D. Revised edition. 8vo, pp. 256. New York: E. B. Treat, 771 Broadway. 1888. (Medical Classics. Price, $2.75.)

A Practical Treatise on Headache, Neuralgia, Sleep and its Derangements, and Spinal Irritation. By J. Leonard Corning, M. A., M. D., Consultant in Nervous Diseases to St. Francis Hospital, etc. 8vo, nearly 300 pages. New York: E. B. Treat, 771 Broadway. 1888. (Medical Classics. Price, $2.75.)

Man a Revelation of God. By Rev. G. E. Ackerman, A. M., M. D., D. D., Member of the American Institute of Christian Philosophy, etc. 8vo, pp. 396. New York: Phillips & Hunt. 1888.

The President's Address. By Robert Battey, M. D., Rome, Ga. Reprint from Vol. XIII., Gynecological Transactions. 1888.

Études Thérapeutiques et Bactériologiques sur le Furoncle de L'Oreille. Par le Docteur Lœwenberg. Extract de L'Union Médicale (3e série). Année 1888. Paris.

Inflation of the Stomach with Hydrogen Gas in the Diagnosis of Wounds and Perforation of this Organ, with the Report of a Case. By N. Senn, M. D., Ph. D., of Milwaukee, Wis., Attending Surgeon to the Milwaukee Hospital, Professor of the Principles and Practice of Surgery and Surgical Pathology in Rush Medical College, Chicago, Ill. Reprint from the *Medical News*, August 25, 1888.

Two Cases of Gunshot Wound of the Abdomen, illustrating the Use of Rectal Insufflation with Hydrogen Gas as a Diagnostic Measure. Same author. Reprint from the *Medical News*, November 10, 1888.

The Radical Cure of Varicocele, attended with Redundancy of the Scrotum, Demonstrated by Time. By Morris H. Henry, M. A., M. D., LL. D., Reprinted from the *Journal of the American Medical Association*, November 10, 1888.

Recent Advances in State Medicine. Annual Address by the Chairman of the Section on State Medicine of the American Medical Association. By Henry B. Baker, M. D., Secretary State Board of Health of Michigan. Reprint from the Journal of the Association, September 22, 1888.

Malaria; and the Causation of Periodic Fever. By Henry B. Baker, M. D., Lansing, Mich. Read in the Section on State Medicine of the American Medical Association at the thirty-ninth annual meeting, May, 1888. Reprint from the Journal of the Association, November 10, 1888.

Relations of Certain Meteorological Conditions to Diseases of the Lungs and Air Passages, as shown by Statistical and other Evidence. By Henry B. Baker, A. M., M. D., Lansing, Mich. Reprint from the Annual Report of the Michigan State Board of Health. 1888.

Alpine Winter, in its Medicinal Aspects; with Notes on Davos Platz, Wiesen, St. Moritz, and the Maloja. By. A. Tucker Wise, M. D., L. R. C. P., M. R. C. S., etc. Fourth edition. London: J. & A. Churchill, New Burlington street, W. 1888.

Diseases of the Nose and Pharynx, and their Treatment. By W. Cheatham, M. D., Louisville, Ky. Reprint from *Virginia Medical Monthly*, December, 1888.

A Digest of the More Important Publications on Sulfonal-Bayer, the new hypnotic. Published by W. H. Schieffelin & Co., New York. 1888.

The History of the Filaria Sanguinis Hominis, its Discovery in the United States, and especially the Relationship of the Parasite to Chylocele of the Tunica Vaginalis Testis. By William M. Mastin, M. D., Mobile, Ala. Reprint from *Annals of Surgery*, November, 1888.

Health Bulletins. Monthly Bulletin New York State Board of Health for October, 1888.

Health in Michigan. November, 1888.

Reports of Deaths and Contagious Diseases, November, 1888, Newport, R. I.

Weekly Abstract of Sanitary Reports U. S. Marine Hospital Service. Abstracts 48, 49, 50, and 51.

Table Talk. Volume III., No. 12. December, 1888. The American authority upon all culinary and household topics. Table Talk Publishing Co., Philadelphia, Pa.

